# Isolated syndrome of inappropriate antidiuresis (SIAD) after Transphenoidal surgery of a non-functioning pituitary adenoma: clinical case report

**DOI:** 10.1093/omcr/omaf152

**Published:** 2025-08-25

**Authors:** Nasthia Quilismal, Mariana Risso, Patricia Agüero, Ramilo Lima, Francisco Garagorry, Dardo Centurion, Maria M Pineyro

**Affiliations:** Unidad Académica de Endocrinología y Metabolismo, Hospital de Clínicas “Dr. Manuel Quíntela”, Facultad de Medicina, Universidad de la República, Avenida Italia S/N, CP 11600, Montevideo, Uruguay; Unidad Académica de Endocrinología y Metabolismo, Hospital de Clínicas “Dr. Manuel Quíntela”, Facultad de Medicina, Universidad de la República, Avenida Italia S/N, CP 11600, Montevideo, Uruguay; Unidad Académica de Endocrinología y Metabolismo, Hospital de Clínicas “Dr. Manuel Quíntela”, Facultad de Medicina, Universidad de la República, Avenida Italia S/N, CP 11600, Montevideo, Uruguay; Unidad Académica de Neurocirugía, Hospital de Clínicas “Dr. Manuel Quintela”, Facultad de Medicina, Universidad de la República, Avenida Italia S/N, CP 11600, Montevideo, Uruguay; Unidad Académica de Anatomía Patológica, Hospital de Clínicas “Dr. Manuel Quintela”, Facultad de Medicina, Universidad de la República, Avenida Italia S/N, CP 11600, Montevideo, Uruguay; Unidad Académica de Anatomía Patológica, Hospital de Clínicas “Dr. Manuel Quintela”, Facultad de Medicina, Universidad de la República, Avenida Italia S/N, CP 11600, Montevideo, Uruguay; Unidad Académica de Endocrinología y Metabolismo, Hospital de Clínicas “Dr. Manuel Quíntela”, Facultad de Medicina, Universidad de la República, Avenida Italia S/N, CP 11600, Montevideo, Uruguay

**Keywords:** syndrome of inappropriate antidiuresis, transsphenoidal surgery, hyponatremia, arginine vasopressin deficiency

## Abstract

**Introduction:**

Disorders of water balance, including arginine vasopressin deficiency (AVP-D) and syndrome of inappropriate antidiuresis (SIAD), are common postoperative complications following pituitary surgery. While AVP-D typically occurs as an isolated condition, SIAD may also present independently.

**Clinical Case:**

We describe the case of a patient with a non-functioning pituitary adenoma who underwent transsphenoidal surgery. On postoperative day 7, she developed isolated SIAD, characterized by a plasma sodium level of 113 mmol/L, normovolemia, urine osmolality of 364 mOsm/kg, and urine sodium concentration of 50 mmol/L. A daily fluid restriction of 800 mL resulted in the resolution of hyponatremia within 48 h.

**Conclusions:**

Water balance disorders following pituitary surgery are often unpredictable. This case highlighs the importance of close postoperative clinical and biochemical monitoring. Vigilance is essential, even in the absence of AVP-D, to ensure early detection and management of potentially severe complications such as SIAD.

## Introduction

Disorders of water balance-such as arginine vasopressin deficiency (AVP-D), formerly known as diabetes Insipidus, and syndrome of inappropriate antidiuresis (SIAD), previously referred to as syndrome of inappropriate antidiuretic hormone secretion- are common complications following pituitary surgery, with reported prevalence rates of 10–30% for AVP-D and 3.6–19.8% for SIAD [1, 2]. These conditions result from alterations in antidiuretic hormone (ADH) secretion [3]. The most frequent disturbances in postoperative water homeostasis include transient AVP-D and biphasic patterns, in which AVP-D is followed by SIAD-induced hyponatremia. Isolated SIAD is less common and usually asymptomatic, presenting as normovolemic hyponatremia [4]. Nevertheless, SIAD is the most common cause of hyponatremia after pituitary surgery [5], with incidence estimates ranging from 1.7% and-23%. Its occurrence is associated with prolonged hospital stays and increased healthcare costs [6]. SIAD most commonly develops approximately one week after surgery, but its onset may range from postoperative day 4 to day 14. We report a case of severe isolated SIAD in a 62-year-old woman following transsphenoidal surgery for a non-functioning pituitary adenoma.

### Clinical history

**Figure 1:**
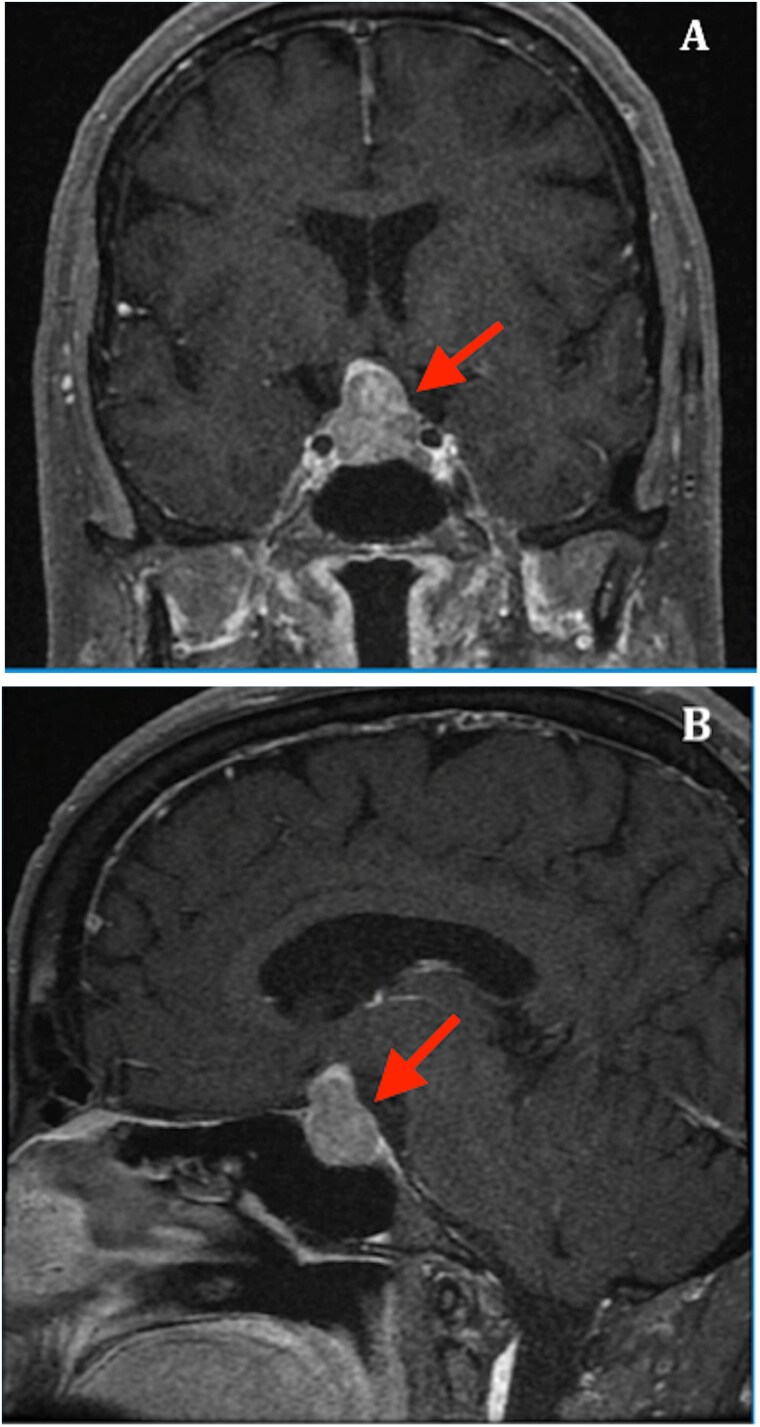
T1-weighted magnetic resonance imaging (MRI) with gadolinium contrast: (A) coronal and (B) sagittal views. The images revealed a pituitary macroadenoma measuring 22 × 18 × 18 mm, exerting superior compression of the pituitary stalk and the optic chiasm.

A 62-year-old female with a history of primary hypothyroidism (treated with 50 mcg of levothyroxine daily) and hyperprolactinemia (prolactin: 172 ng/ml; reference range: 4.8–23.3 ng/ml) presented without headaches and visual disturbances. The remaining pituitary hormonal axes were normal. Magnetic resonance imaging revealed a 22 × 18 × 22 mm pituitary macroadenoma compressing the optic chiasm, with no visible pituitary stalk (Fig. 1). A formal visual field test showed no abnormalities. A diagnosis of non-functioning pituitary adenoma was made, and the patient underwent an uneventful transsphenoidal resection. The immediate postoperative period was unremarkable, and no evidence of AVP-D was observed.

On postoperative day 7, she reported new-onset headache and asthenia, with no other symptoms. She remained clinically euvolemic. Laboratory testing revealed a plasma sodium level of 113 mmol/l, urine osmolality of 364 mOsm/kg, urine sodium concentration of 50 mmol/l, calculated plasma osmolality of 244, 3 mOsm/kg, and normal renal function test. She was no taking diuretics. At the time, she was on hydrocortisone replacement (10 mg at 8:00 AM and 5 mg at 4:00 PM) due to a postoperative morning cortisol level of 10 μg/dl.

These findings were consistent with the syndrome of inappropriate antidiuresis. Fluid restriction to 800 ml/day led to normalization of sodium levels to 143 mmol/l within 48 h. Histopathology analysis confirmed a gonadotroph adenoma, with immunopositivity to FSH and LH.

## Discussion

Isolated SIAD is a rare postoperative complication, reported in approximately 5–9% of cases following pituitary surgery [7].

Risk factors for delayed hyponatremia include older age, low body mass index, preoperative hyponatremia or hypopituitarism, optic chiasm compression, Cushing’s disease, early postoperative AVP-D, cerebrospinal fluid leakage, a decline in sodium on postoperative day 1, and prolonged surgery duration [5, 6]. Although SIAD risk may theoretically increase with macroadenomas compressing the pituitary stalk due to disruption of hypothalamic pathways, a study of over 1000 lesions found no association between tumor size or location and postoperative hyponatremia [6].

SIAD results from neuronal damage to the hypothalamo-pituitary tract, leading to delayed ADH release [5]. In Isolated SIAD, partial injury to the pituitary stalk may spare some vasopressin-producing neurons, preventing the development of complete AVP-D.

Hyponatremia due to SIAD leads to hypoosmolality and cellular edema, which can result in neurological symptoms. Mild cases are often asymptomatic, whereas severe cases may present with headache, nausea, vomiting, irritability, confusion, impaired concentration, stupor, seizures, or coma [5].

Monitoring of serum sodium levels between postoperative days 5–7- and up to day 15 if symptoms arise- is essential to detect delayed SIAD [8].

The diagnostic criteria for SIAD include: plasma sodium < 135 mmol/l, plasma Osmolality < 275 mOsm/kg, urinary Sodium > 30 mmol/l, urinary Osmolality > 100 mOsm/kg, and euvolemia. Our patient met all these criteria. Furthermore, adrenal insufficiency and hypothyroidism must be excluded. In this case, hydrocortisone was initiated postoperatively due to a morning cortisol of 10 μg/dl.

The differential diagnosis of SIAD includes hypotonic fluid overload, medication-induced hyponatremia (e.g. diuretics, SSRIs, NSAIDs, antipsychotics), desmopressin overuse, and hyperglycemia [5].

Cerebral salt wasting syndrome (CSWS) must be also considered. The key difference is that it causes hypovolemia due to natriuresis and aldosterone suppression, whereas SIAD is euvolemic (Table 1). CSWS treatment is fluid and sodium replacement, while SIAD treatment is fluid restriction [5].

**Table 1:** Differences between SIAD and CSWS.

Feature	SIAD (Inappropriate ADH secretion)	CSWS (Cerebral Salt Wasting)
Pathophysiology	Excessive ADH secretion causing renal water retention	Renal sodium loss due to impaired tubular sodium reabsorption and elevated natriuretic factor
Plasma Volume Status	Normal or slightly increased (euvolemic)	Decreased (hypovolemic)
Urine Sodium (Na)	Elevated (>40 mmol/l)	Elevated (>40 mmol/l), often markedly increased (>100 mmol/l)
Urine Osmolality	Inappropriately elevated (>100 mOsm/kg)	Elevated
Blood Urea	Low	Elevated
Clinical Signs of Volume Status	No clinical signs of hypovolemia	Signs of volume depletion (hypotension, orthostasis, tachycardia, decreased skin turgor)
Hematocrit and Plasma Proteins	Normal or low (dilutional effect)	Increased due to hemoconcentration
Brain Natriuretic peptide	Normal	Increased
Treatment	Fluid restriction, consider hypertonic saline cautiously; Vasopressin receptor antagonists (in selected cases)	Volume repletion with isotonic or hypertonic saline

Management of SIAD depends on the severity and acuity of hyponatremia and the presence of neurological symptoms. Hyponatremia is classified as mild (131–134 mmol/l), moderate (125–130 mmol/l), or severe (<125 mmol/l).

Acute (<48 h) and severe hyponatremia typically presents symptomatically. First-line treatment is fluid restriction (500–1000 ml/day), which was effective in our patient [5].

If fluid restriction is insufficient, additional strategies include increased solute intake (protein or salt) to enhance water excretion, administration of urea (oral or IV) as a non-reabsorbable urinary solute, and use of vasopressin receptor antagonists (vaptans) [9]. Tolvaptan, a selective V2 receptor antagonist, with a starting dose at 15 mg/day and may be titrated to 60 mg/day. It increases serum sodium by approximately 5 mEq/l after 4 days. Limitations to its use include hepatotoxicity and high cost [9].

Rapid correction of hyponatremia may result in osmotic demyelination syndrome [5].

Safe correction targets recommend an increase serum sodium of 4–6 mEq/l within 24 h, with a maximum of 8 mEq/L per day when serum sodium is < 120 mEq/l [9].

Predicting postoperative AVP-D or SIAD remains challenging due to sodium level fluctuations. Given the average 3-day hospital stay following pituitary surgery, delayed hyponatremia may manifest at home. Some studies suggest that prophylactic fluid restriction may reduce readmission rates [5]. A meta-analysis by Yu et al. found that postoperative fluid restriction protocols decreased the incidence of late-onset hyponatremia (OR 5.02; CI 95%: 2.16–11.65). However, heterogeneity in restriction protocols limits widespread applicability [10].

## Conclusion

Disorders of water balance are frequent complications following pituitary surgery. Isolated SIAD may develop independently, even in the absence of AVP-D. Given the unpredictability nature of these conditions, vigilant postoperative monitoring is essential to promptly detect and manage delayed-onset hyponatremia, which can be potentially life-threatening.

## Contributions

NQ wrote the first draft of the manuscript. MP contributed to the writing of the manuscript. NQ, MR, PA, RL, FG, DC and MP made contributions to the acquisition of the clinical data, agreed with manuscript results and conclusions. NQ and MP made critical revisions and approved final version. All the authors revised and approved the final manuscript and agreed to be accountable for the content of the work.
